# MICAR: multi-inhabitant context-aware activity recognition in home environments

**DOI:** 10.1007/s10619-022-07403-z

**Published:** 2022-04-05

**Authors:** Luca Arrotta, Claudio Bettini, Gabriele Civitarese

**Affiliations:** EveryWare Lab, Department of Computer Science, University of Milan, Milan, Italy

**Keywords:** Activity recognition, Multi-inhabitant, Smart-home, Semi-supervised learning

## Abstract

The sensor-based recognition of Activities of Daily Living (ADLs) in smart-home environments enables several important applications, including the continuous monitoring of fragile subjects in their homes for healthcare systems. The majority of the approaches in the literature assume that only one resident is living in the home. Multi-inhabitant ADLs recognition is significantly more challenging, and only a limited effort has been devoted to address this setting by the research community. One of the major open problems is called *data association*, which is correctly associating each environmental sensor event (e.g., the opening of a fridge door) with the inhabitant that actually triggered it. Moreover, existing multi-inhabitant approaches rely on supervised learning, assuming a high availability of labeled data. However, collecting a comprehensive training set of ADLs (especially in multiple-residents settings) is prohibitive. In this work, we propose MICAR: a novel multi-inhabitant ADLs recognition approach that combines semi-supervised learning and knowledge-based reasoning. Data association is performed by semantic reasoning, combining high-level context information (e.g., residents’ postures and semantic locations) with triggered sensor events. The personalized stream of sensor events is processed by an incremental classifier, that is initialized with a limited amount of labeled ADLs. A novel cache-based active learning strategy is adopted to continuously improve the classifier. Our results on a dataset where up to 4 subjects perform ADLs at the same time show that MICAR reliably recognizes individual and joint activities while triggering a significantly low number of active learning queries.

## Introduction

Assisted living technologies based on ambient intelligence are becoming a fundamental tool to continuously monitor and assist fragile elderly subjects in their homes, also considering the recent Covid-19 pandemic [40]. Among the many advantages of such systems (e.g., alarms, medication reminders, home automation), they can also support clinicians’ diagnoses. For instance, such technologies have been proposed to detect early symptoms of cognitive impairments [42]. In order to detect complex human behaviors, the sensor-based recognition of Activities of Daily Living (ADLs) is a fundamental step [6].

ADLs recognition in smart-home environments has been extensively studied in the last decades [11]. Due to privacy reasons, those approaches are generally based on environmental or wearable sensors to monitor residents’ high-level activities like cooking, taking medicines, or watering plants. Indeed, solutions based on microphones or cameras are usually considered as intrusive for a home environment, especially considering elderly subjects.

The majority of existing ADLs recognition methods tackled single-inhabitant settings, where only one resident lives in the home. Those scenarios are realistic, considering that a significant number of elderly subjects live alone in their apartments. However, it often happens that multiple residents live in the same home (e.g., a couple of elderlies, an elderly and the caregiver, a family, etcetera). In order to accurately detect ADLs for the fragile target subjects, it is crucial to correctly discriminate the activities performed by each resident. Differently to single-inhabitant settings, multiple residents may perform ADLs jointly (e.g., Alice and Bob are cooking together) and concurrently (e.g., Alice watches TV while Bob is cooking).

Recently, several research efforts on multi-inhabitant ADLs recognition have been proposed in the literature [31]. The major open research problem in this area is that environmental sensor events (e.g., the opening of a kitchen drawer revealed by a magnetic sensor) do not directly identify the resident who triggered it. Indeed, without supplementary hardware, environmental sensors merely reveal state changes. This problem is referred to as *data association*: mapping each environmental sensor event to the inhabitant which triggered it [7].

Another well-known problem is labeled data scarcity. Collecting an annotated dataset of ADLs in a multi-inhabitant setting is time-consuming, costly, and intrusive. A comprehensive labeled multi-inhabitant dataset should cover all the possible combinations of ADLs performed by the residents, also considering every possible execution modality. While existing multi-inhabitant solutions assume complete availability of labeled data [31], we believe that this is a relevant problem that should be addressed in the literature.

Semi-supervised learning is a possible solution to mitigate the data scarcity problem. According to this approach, the recognition model is initialized with a limited amount of labeled data, while unlabeled data are exploited to improve the classifier. Semi-supervised solutions for activity recognition are recently emerging [10] but, to the best of our knowledge, there is no existing work that proposes semi-supervised learning for multi-inhabitant ADLs recognition.

In a previous work [3], we proposed a multi-inhabitant ADLs recognition framework that tackled the data association problem using context-aware reasoning. However, that approach was based on supervised learning, thus assuming full availability of labeled data. Moreover, that work did not propose a method to identify group activities.

In this paper, we extend our previous work by presenting MICAR: a novel Multi-Inhabitant semi-supervised and Context-aware Activity Recognition framework. Our approach combines wearable and environmental sensors data to derive high-level context information (e.g., residents’ posture, residents’ location) to reliably perform data association using a semantic-based approach. MICAR tackles the labeled data scarcity problem thanks to a novel cache-based active learning approach to continuously improve the classifier (initialized with limited labeled data) while triggering a limited number of questions. MICAR is capable of detecting both individual and group ADLs.

We extensively evaluated MICAR on a new dataset where up to four residents perform ADLs collaboratively or individually. Our results indicate that MICAR reaches a high recognition rate (F1-score $\approx 0.89)$ that is slightly behind a fully supervised approach while triggering a low number of active learning queries (query rate $\approx 3\%$). Moreover, our results confirm that our context-aware data association leads to a recognition rate that is only $2\%$ behind the one obtained by an ideal approach based on ground truth. Our results also indicate that MICAR is accurate in detecting the number of users that jointly perform an ADL.

The contributions of this paper are the following:

- We introduce MICAR: a novel hybrid semi-supervised and context-aware multi-inhabitant activity recognition method that combines knowledge-based and data-driven approaches.
- MICAR  tackles the data association by relying on a novel semantic approach
- MICAR takes advantage of a novel cache-based active learning approach to mitigate the labeled data scarcity problem.
- We performed an extensive evaluation on a multi-inhabitant dataset where up to 4 residents live in the same home. Our results show that MICAR  reaches recognition rates comparable to the ones of a fully supervised approach and that the accuracy of data association is close to the one of an ideal approach based on ground truth.

The rest of the paper is organized as follows. Section 2 discusses the relevant literature on multi-inhabitant activity recognition and semi-supervised learning. Section 3 formally describes the multi-inhabitant activity recognition problem. Section 4 describes the overall architecture of MICAR. Section 5 describes each component of MICAR in detail. Section 6 describes the dataset, the evaluation methodology, and the main results. Finally, Sect. 8 concludes the paper.

## Related work

### Multi-inhabitant activity recognition

The majority of the literature on sensor-based ADLs recognition mainly focused on smart-home environments inhabited by a single person [11, 15], while fewer research efforts investigated multi-inhabitant scenarios [7, 31]. Several works proposed solutions based on computer vision [27]. However, cameras are perceived as too privacy-intrusive considering home environments. For this reason, MICAR (in line with the recent literature) focuses on wearable and environmental sensors.

A challenging problem in sensor-based multi-inhabitant ADLs recognition is that an environmental sensor event does not identify the resident that triggered it. For instance, a pressure mat sensor on a chair cannot reveal the residents sitting on it. The process of mapping environmental events to the correct resident is called *data association*, and it is essential to reliably infer the activities performed by each inhabitant [7].

In the literature, multi-inhabitant ADLs recognition has been tackled with different approaches. We divide those approaches in three categories: *single-model*, *data-driven data association*, and *wearable-based data association*.

The *single-model* approaches rely on a single ADLs classifier that directly attributes activities to users based on the stream of raw environmental sensor data. Hence, data association is implicitly learned during the training phase. These approaches encode the residents’ identifiers in the activity labels [1, 2, 8, 21, 35, 36, 47]. The main drawback of *single-model* methods is their poor scalability. Indeed, training data should contain examples for all the possible combinations of activities that the residents can potentially perform together or individually. The learning complexity significantly increases with the number of residents and the number of activities. At the same time, it is even more challenging to transfer a multi-inhabitant ADLs model to different environments/residents than a single-inhabitant one. MICAR  takes advantage of data association to use a single-inhabitant classifier for each user. Single-inhabitant models are easier to train, and their transferability is well-studied in the literature [16]. We also mention that there are *single-model* approaches that do not identify the specific resident that performed each activity, only focusing on discriminating concurrent activities [39].

The *data-driven data association* approaches consider data association as a separate learning problem before ADLs classification. In [17], labeled data about behaviors and habits of the residents are used to train a supervised classifier that attributes a resident to each sensor event. Another work is based on a multi-target Gaussian mixture probability hypothesis density (GM-PHD) filter that learns the Spatio-temporal relationships among the environmental sensors’ events to identify the residents that triggered them [48]. Besides supervised solutions, *data-driven data association* was tackled with unsupervised learning [41]. That method relies on mining single-inhabitant unlabeled datasets to perform a weaker form of data association, called *resident separation*. A *resident separation* model identifies in real-time the pairs of environmental sensors’ events triggered by the same resident. The main problem of *data-driven data association* approaches is that they are heavily based on the specific environment and on the habits of the residents used to create the data association model. Hence, their generalization capability is questionable. As we mentioned in the introduction, data scarcity is an open problem in activity recognition. Hence, requiring data also to train a data association model is even more challenging considering real-world deployments. MICAR  relies on a semantic approach for data association based on context data. Hence, no data is required to learn how to associate environmental events with residents.

The *wearable-based data association* approaches require each resident to wear supplementary sensors (e.g., wristbands, smartphones) to correctly associate environmental events. MICAR falls in this category. In [49], the residents wear a specifically designed RFID wristband reader in charge of detecting the interaction with tagged objects. However, the use of sophisticated wearable devices in realistic deployments is questionable, especially considering elderly subjects that should constantly wear them. On the contrary, MICAR relies on off-the-shelf smartwatches. We indeed believe that smartwatches represent non-intrusive devices that are commonly used in the last years, and they also have been deeply studied in the literature for elderly subjects in their homes [32]. Recent research works in geriatric nursing confirm that activity trackers like wristbands and smartwatches are perceived as acceptable by elderly subjects [37]. We also believe that, in the near future, advances in miniaturization and micro-localization (e.g., small tags based on Ultra Wide Band like the AirTag proposed by Apple) will be available to enable context-aware data association with more unobtrusive solutions. For instance, as hypothesized in the literature, micro-localization devices could be miniaturized and equipped in everyday clothes (e.g., slippers) [24].

Another work proposed a solution to track and identify people in a multi-resident setting using personal devices [29]. The authors propose a combination of PIR sensors and Bluetooth Low Energy (BLE) beacons deployed in the home. The RSSI signal from BLE beacons is exploited to associate PIR events with the closest resident, relying on personal wearable devices like a smartwatch, smartphone, or wristband. However, while that work is only limited to residents tracking and identification, MICAR  uses micro-localization in combination with other context data to perform data association considering a wide variety of environmental sensors to support multi-inhabitant ADLs recognition. Finally, a closely related work combines micro-localization and smartphone sensors to detect group activities [13]. In that approach, acceleration, location, and audio signals are combined to understand if multiple subjects are performing the same activity. However, that approach does not include environmental sensors and it is designed to capture a few group activities that are very distant from our target ADLs (e.g., taking a class, having a discussion).

### Semi-supervised approaches for ADLs

The recognition of ADLs has been mainly addressed with solutions based on supervised machine learning [12]. The major drawback of those approaches is that they require a large amount of labeled data to train the activity models. Among the many proposals in the literature to tackle the data scarcity problem, semi-supervised learning approaches for ADLs recognition represent a promising research direction [46]. Semi-supervised models are initialized with a limited amount of labeled data, and the stream of unlabeled data is annotated using techniques like label propagation [45] or active learning [26].

In the literature, *active learning* is one of the most commonly adopted semi-supervised approaches for ADLs recognition, due to its significant impact on the recognition rate [5, 14, 23, 25, 26]. In particular, when the activity classifier is uncertain on the current prediction, a query is prompted to the user to obtain the ground truth about the activity that she is performing. The feedback is used to improve the recognition model. In [9] we also proposed a hypothetical active learning interface for multi-inhabitant settings. However, that work did not propose a practical semi-supervised data association and multi-inhabitant activity recognition methodology.

Other semi-supervised approaches like label propagation and co-training have also been investigated for ADLs recognition [20, 22, 34, 50]. Co-training combines the output of multiple classifiers that have been trained on different views of the training data [33]. Label propagation takes advantage of the few available labels to associate labels to unlabeled data using a graph representation of available data points [45].

To the best of our knowledge, semi-supervised approaches for ADLs recognition have been proposed only for single-inhabitant settings. MICAR  is a semi-supervised ADLs method for multi-inhabitant scenarios, implementing a novel active learning approach that uses a cache to significantly reduce the number of triggered queries.

## The data association problem

Given a limited amount of labeled data, the objective of the activity recognition system (named just *system* in the following) is to periodically infer for each user the activity of daily living (ADL) that she has been performing. The system also detects situations where ADLs are performed in cooperation by multiple inhabitants. Intuitively, a set of users is jointly performing an ADL when those users are in the same place and, according to the system predictions, they are performing the same ADL^1^.

Let ${\mathbf {U}} = \{u_1, u_2, \dots , u_n\}$ be the set of users (the smart-home residents) and ${\mathbf {A}} = \{A_1, A_2, \dots , A_k\}$ the set of target ADLs. Given an instant *t*, the system predicts for each user the activity prediction $\langle u,A,L,t \rangle$, where *u* is the user that performed activity *A* in the semantic location *L*. Hence, the system returns a set of tuples $PA_t=\{ \langle (u_r,\ldots , u_s), A_i, L_j\rangle | \langle u,A_i,L_j,t \rangle \forall u \in (u_r,\ldots , u_s) \}$. Each tuple represents the set of users that jointly performed $A_i$ the same ADL in the same semantic location $L_j$.

In order to achieve this goal, the system continuously analyzes a stream of time-stamped events coming from inertial and environmental sensors. Given an instant *t* and a user *u*, the system needs to solve a *data association problem* to derive a personalized stream $s(u)^t$ of sensor events associated with user *u* and collected in a time window $[t, t-k]$ where *k* is the window size parameter. For example, suppose that Anna opens the fridge door at time $t'$. The corresponding sensor event (and its timestamp) generated by the magnetic sensor connected to the fridge door and recorded by our system should be associated with Anna and hence considered part of $s(Anna)^t$ when $0 \le t-t^{\prime } \le k$.

The data association problem is straightforward for events coming from inertial sensors on personal devices, but challenging for environmental sensors.

## MICAR’s architecture

**Fig. 1:**
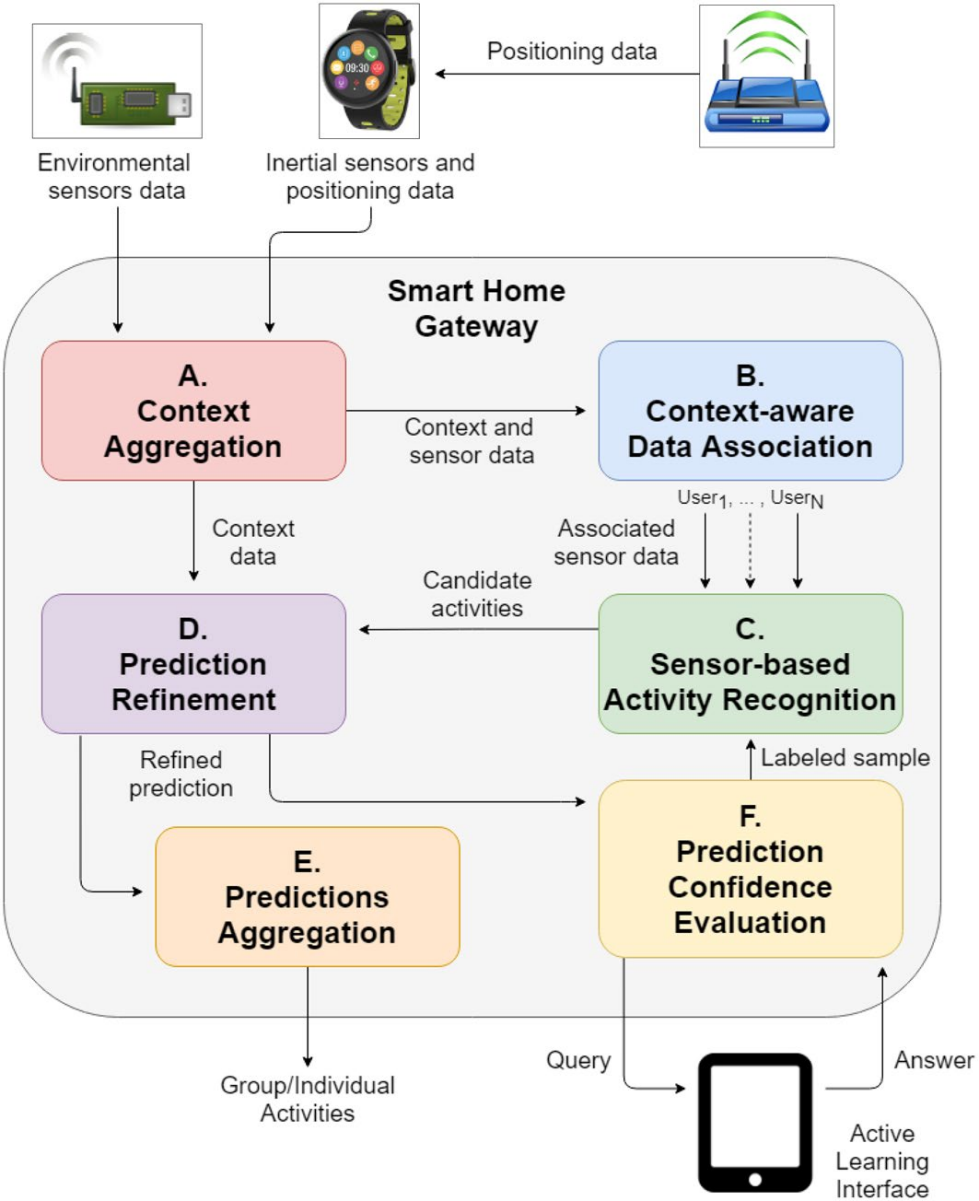
Overall architecture of MICAR

The general architecture of MICAR is depicted in Fig. 1.

Several environmental sensors are deployed in the home (e.g., plug sensors, magnetic sensors, motion sensors) to capture the interaction of the residents with the surrounding environment. Moreover, each resident wears a smartwatch that collects data from its inertial sensors (e.g., accelerometer) and a micro-localization system (e.g., BLE beacons, WiFi) deployed in the environment. Raw sensor data are continuously transmitted to the Smart Home Gateway, which is in charge of running the algorithms of MICAR.

First, the Context Aggregation module pre-processes sensor data to infer higher-level context information (e.g., the residents’ locations, low-level activities, etcetera). High-level context, as well as raw sensor data, are then transmitted to the Context-Aware Data Association module. This module relies on semantic reasoning on context and sensor events to generate a personalized stream of inertial and environmental sensor data for each user. The rationale is that it is possible to use common-sense knowledge in the activity domain to exploit context data to derive the most likely correspondence between each environmental sensor event and the resident that triggered it.

Each personalized stream is then processed by the Sensor-based activity recognition module. This module relies on an incremental semi-supervised classifier to detect the ADLs performed by a specific resident. The output of the classifier is a probability distribution over the possible activities. The recognition model is initialized with a limited number of labeled data from a few users (e.g., 2 in our experiments) that in an initial phase contributed to a small labeled data acquisition campaign.

High-level context is then processed again by the Prediction refinement module to refine the machine learning classification. Indeed, ADLs associated with a positive probability but that contrast with the current context (e.g., watching TV when the TV is not turned on) are removed from the probability distribution.

The Prediction confidence evaluation module evaluates the uncertainty of the refined prediction. If the uncertainty is greater than a threshold, an active learning process is started: the system triggers a query to ask the user which is the activity she is performing through a dedicated interface. The feedback is used to update the incremental activity recognition classifier. Our active learning method is based on a cache to reduce the number of triggered questions.

In parallel to Prediction confidence evaluation, the Predictions Aggregation module combines the refined predictions from each user to output both individual and joint activities performed by the residents. In particular, a heuristic method determines whether multiple residents are performing the same activity.

In the next section, we describe each component of MICAR in detail.

## MICAR under the hood

### Sensing sources

The residents are monitored with a combination of wearable and environmental sensors. In particular, each inhabitant wears a smartwatch, equipped with inertial sensors (e.g., accelerometers, gyroscopes, and magnetometers) to track her physical movements. Inertial sensors are particularly useful to capture ADLs that are characterized by specific gestures (e.g., washing dishes). Smartwatches also collect data (e.g., RSSI) from a positioning system deployed in the home (e.g., BLE beacons, Ultra-Wideband, WiFi access points). Positioning data is particularly useful to continuously monitor the semantic position of the user. Since the smartwatch is a personal device, the collected data can be automatically associated with the resident’s identity.

Environmental sensors capture the interaction of the residents with home infrastructure. For example, magnetic sensors detect the opening and closing events of doors and drawers, pressure mats on chairs reveal if someone is sitting, and smart plugs detect the usage of home appliances. As we already mentioned, environmental sensors cannot identify the resident which triggers them since they only output their status.

### Context aggregation

The Context aggregation module receives the raw data from the sensing sources described above. The objective of this module is to derive higher-level context information. As we described in Sect. 4, MICAR uses high-level context to compute data association as well as to refine the classifier’s prediction.

The Context aggregation module derives the *personalized context* for each resident and the *home context* for the home environment. Given a time instant *t*, the *personalized context* of a resident *u* is denoted with $C(u)^t = (l(u)^t, p(u)^t)$, where $l(u)^t$ is the location of *u* in the home at time *t* and $p(u)^t$ is the posture of *u* at time *t*. For instance, if Bob is sitting in the kitchen at time *t* then $C(Bob)^{t} = (\textit{kitchen}, \textit{sitting})$. On the other hand, the * home context *$C_H^t$ encodes the status and the position of each sensor in the home. In the following, we describe how *C*(*u*) and $C_H$ are computed from raw sensor data.

#### Resident’s semantic position

In the following, we describe how we derive the semantic position $l(u)^t$ of a resident *u* at time *t*. In our implementation, the smartwatch is in charge of collecting RSSI data from a positioning infrastructure composed of a combination of BLE beacons and WiFi access points. Raw RSSI data are segmented with a sliding window of size $n_l$ and overlap $p_l$. Then, we apply a Savitzky-Golay filter to smooth raw RSSI data. In our experimental setup we use $n_l = 5s$ and $p_l = 50\%$.

For each temporal window, we extract a feature vector, where each feature encodes the mean RSSI signal of the window from a specific source (i.e., a specific BLE beacon or WiFi access point). In our experimental setup, the *mean* was sufficient to characterize each signal, while the use of other statistical properties did not lead to any improvement in the positioning accuracy. Finally, a machine learning classifier is in charge of classifying the semantic position of the user from the feature vectors. In our experiments, we used a Random Forest classifier.

Note that the organization of the home in semantic positions should be performed in an offline phase, and its granularity depends on the accuracy of the underlying micro-localization system. A coarse granularity may consider room-level semantic positions (e.g., living room, kitchen, dining room), while a fine-grained granularity may map specific regions of each room into semantic positions (e.g., cooking area, dining table, and sink area).

In our experimental setup, we implemented a micro-localization infrastructure at room-level granularity based on a combination of 5 BLE beacons^2^ uniformly installed within our smart-home lab and 26 WiFi access points that could be detected in its surroundings. Our infrastructure reaches an average positioning error of 1–2 m. However, we did not consider these results to be satisfactory for an accurate data association.

In the literature, several solutions have been proposed for more accurate indoor positioning [51]. MICAR is agnostic to the specific micro-localization system being used, and we preferred to use ground truth information about positioning data in our experiments, in order to focus on multi-inhabitant activity recognition only. We expect that new technologies (e.g., UWB) will be significantly more accurate in indoor localization, and MICAR could adopt them to perform reliable data association.

#### User’s posture

The posture $p(u)^t$ (e.g., standing, sitting, lying) of a user *u* at time *t* is derived by feeding a machine learning classifier with the inertial sensors data from the smartwatch. First, we pre-process raw data by applying a median filter to reduce the noise. Then, we apply sliding window segmentation, with a window size of $n_p$ seconds windows and overlap $p_p$. In our experimental setup we use $n_p = 8s$ and $p_p = 80\%$. For each temporal window, we extract several features that are well-known to be accurate for activity recognition [30]. We obtain in total 120 inertial features, which are then dimensionally reduced to $d_p$ values through the ANOVA technique [44], and finally standardized. In our experiments, we determined $d_p = 84$. Each feature vector is provided to a machine learning classifier to distinguish between different postures. In our experiments, we used a fully connected neural network to discriminate between *sitting* and *not sitting*.

#### Sensor status and position

As we previously mentioned, MICAR also computes $C_H^t$ as the context of the home environment. An important contextual aspect is the semantic position of each sensor, which we consider as prior knowledge defined during the deployment phase in the smart-home. During the deployment phase, we also map each environmental sensor to a semantic concept. For instance, when the magnetic sensor installed on the fridge door fires, it generates the high-level event $(\textit{fridge\_door}, \textit{kitchen}, \textit{OPEN})$, which means that the fridge door in the kitchen has been opened.

$C_H^t$ keeps track of the current status of environmental sensors by considering the previously mentioned high-level information.

##### Example 1

Consider a home *H* equipped with two plug sensors: one to detect the usage of the electrical stove in the kitchen and one to detect the usage of the television in the living room. Suppose that at time *t* Bob is watching TV and that no one is using the electrical stove. In this case $C_H^t = \{( \textit{stove,kitchen,OFF} ), (\textit{television}, \textit{living}\_\textit{room}, \textit{ON})\}$.

### Context-aware data association

Given the high-level context from Context aggregation and the raw sensor data collected from inertial and environmental sensors, the goal of data association is to periodically compute for each user *u* a personalized sensor data stream $s(u)^t$. A stream $s(u)^t$ consists of the inertial sensor readings gathered from the personal device of *u*, and the environmental sensor events triggered by *u* in a time window $[t, t-k]$, where *k* is the size of the segmentation window. Note that $s(u)^t$ is computed every time a new environmental sensor event (*e*, *st*, *t*) occurs. In our experiments, we empirically determined $k=14s$.

As we previously mentioned, the challenge of data association is to assign environmental sensor events to the resident that most likely triggered it. Indeed, an environmental sensor event (*e*, *st*, *t*) (e.g. $(\textit{fridge}\_\textit{door}, \textit{OPEN}, \textit{12:32})$) cannot directly identify the user who triggered it.

MICAR performs data association by exploiting the high-level context data. In particular it approximates a stream $s(u)^t$ by including all the environmental events that are *consistent* with $C(u)^t$ and $C_H^t$. The notion of *consistency* is inherently related to the semantics of the context and the action revealed by the event. The Context-Aware Data association module of MICAR is implemented with ontological reasoning. In particular, an OWL2 ontology defines the relationships between environmental sensor events and contexts. In the following, we describe some axioms that we encode in our ontology.

Among other constraints, our ontology imposes that a user can trigger a sensor event only if she is in the same *semantic position* where the sensor is located (e.g., Alice cannot turn on the TV in the living room while she is in the bedroom). Other axioms combine *user’s posture* and *sensor status and position* to better associate environmental sensor events when multiple users are in the same semantic position at the same time. For instance, the activation of the pressure mat can be associated only with those users which recently switched to the *sitting* posture. Similarly, the *sitting* posture is not compatible with sensor events that can be triggered only while *standing* (e.g., turning on the stove).

In general, when a sensor event $(e,st,t^{\prime })$ is triggered, our system checks its context-consistency for each user *u* using ontological reasoning. In particular, MICAR adds factual observations to the ontology to describe the sensor event, the context $C(u)^{t^{\prime }}$, and the context $C_H$. Then, by using the automatic consistency check of the resulting ontology, the system decides whether $(e,st,t')$ should be included in $s(u)^t$ (with $t^{\prime }$ in the time window defined by *t*)

The output of Context-Aware Data Association is hence a personal stream $s(u)^t$ $\forall u \in {\mathbf {U}}$. The solution is approximate since there may not be sufficient information to associate an event to a single user and, in this case, the event will be associated with the stream of each candidate user.

#### Example 2

Suppose that Alice and Bob are both in the kitchen, and the magnetic on the fridge generates an event at time *t*, thus indicating that someone opened it. Suppose that Alice is standing, while Bob is sitting on a chair. Those context information are detected by the Context Aggregation module. Hence, MICAR adds to the ontology the observations about the residents in the home (i.e., Anna and Bob), their high-level context (i.e., Anna is standing in the kitchen, Bob is sitting in the kitchen), and the triggered environmental sensor event (the fridge magnetic sensor is ON). By performing a consistency test, our ontology derives that the opening fridge event is consistent with Alice’s context, while it is not consistent with Bob’s context (i.e., a user cannot open the fridge if he is *sitting*). Hence, in this case, the fridge event will be included in $s(Alice)^t$ and not in $s(Bob)^t$.

**Fig. 2:**
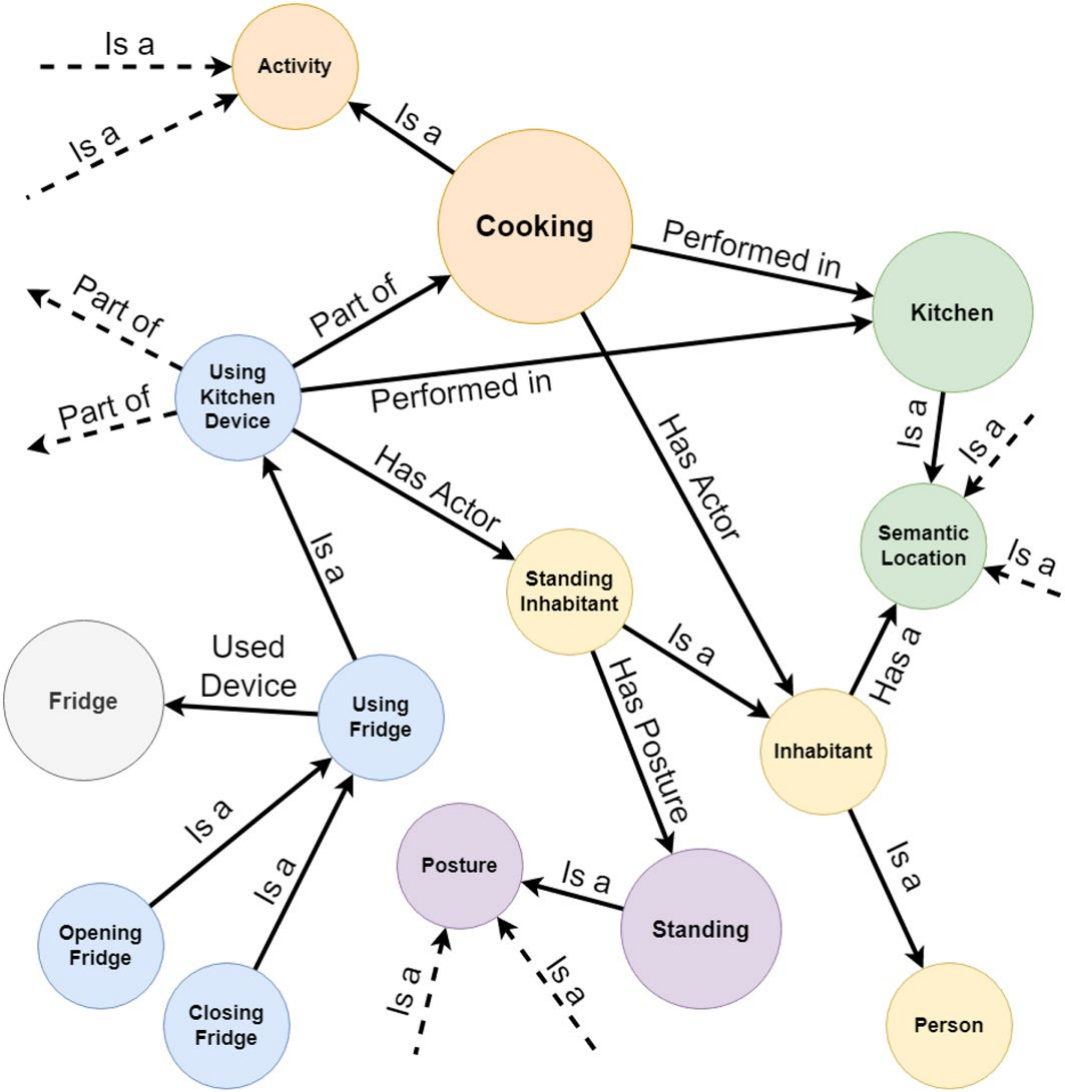
A simplified representation of a small portion of our ontology. Each node encodes an entity, while each edge encodes a relationship

We show a small sample of our ontology in Fig. 2. In order to simplify the visualization, the ontology is represented as a graph where each node is an entity, while each edge encodes a relationship.

### Sensor-based activity recognition

The objective of the Sensor-based activity recognition module is to infer the activities performed by each user in the home. For each resident *u*, it periodically processes the personalized stream $s(u)^t$ received from Context-Aware Data Association to derive the activity performed for *u* at time *t*.

#### Segmentation and feature extraction

MICAR considers each personalized $s(u)^t$ as a temporal window of size *k*. In order to improve the recognition model, we also compute overlapping segmentation between consecutive windows considering an overlap factor of $p_{ar}$. In our experiments, we determined $p_{ar}=80\%$.

From each segmentation window, MICAR extracts different features from inertial and environmental sensors data. Considering inertial data, we apply a median filter for noise reduction. Hence, we extract 120 well-known statistical features from accelerometer, gyroscope, and magnetometer data [30]. Examples of such features are: *root mean square*, *kurtosis*, *symmetry*, *zero-crossing rate*, *number of peaks*, and *energy*, and the *Pearson correlation*. Considering environmental sensors data, we extract 36 features that are based on the status of the smart-home sensors and the number of their activation and deactivation events. In particular, MICAR implements the feature extraction technique based on temporal decay that was proposed in [28]. In our experiments, we applied ANOVA to reduce the dimensionality, reducing the feature space from 156 features to 84.

#### Activity recognition

Each feature vector *fv* generated from the personalized stream of a resident *u* is provided to an incremental single-inhabitant ADLs classifier *h* to derive the probability distribution over the possible ADLs performed by *u*:

$$\begin{aligned} h(fv) = \langle p_{A_1}, p_{A_2}, \dots , p_{A_n} \rangle \end{aligned}$$

where $p_{A_i}\in [0,1]$ $\forall i$, $\sum _{i=1}^n p_{A_i} = 1$, and $p_{A_i}$ is the probability $P(A_i | fv)$ that the resident *u* is performing activity $A_i \in {\mathbf {A}}$, based on *fv*. Note that the activity recognition classifier is initialized using a limited amount of labeled data from a restricted number of users. MICAR does not impose a specific choice for the single-inhabitant classifier.

In our experiments, we implemented a Deep Neural Network which combines convolutional (CNN) and recurrent (LSTM) layers, inspired by the work proposed in [38]. Our network is composed of a 1D CNN layer with 256 filters, followed by a 256 units LSTM layer. The CNN layer is in charge of extracting features in the latent space that better represent input data, while the LSTM layer captures the temporal relationships between segmentation windows. Then, the network continues with a fully connected layer of 128 neurons. Finally, a softmax layer generates the probability distribution over the possible activities. Note that, for the sake of regularization, all the layers are separated by a dropout layer (with a dropout rate of 0.5). The network architecture was determined empirically. Its simplicity is due to the reduced dimensionality of the input feature space. Indeed, we experimented with more complex networks that did not lead to significant improvements in the recognition rate.

### Prediction refinement

Activity recognition classifiers are sometimes not accurate, confusing ADLs that share similar sensor patterns. Considering machine learning-based approaches, the training set is often limited and it may not generalize on unseen activity patterns. As a drawback, the classifier can potentially derive a wrong activity. However, common-sense knowledge about the relationships between activities and context can be used to mitigate those classification mistakes.

MICAR uses the high-level context $C(u)^t$ and $C_H^t$, computed by the Context Aggregation module, to refine each activity prediction *h*(*fv*). In particular, MICAR adopts an approach inspired by the one proposed in [10]. The Prediction refinement module applies knowledge-based reasoning on context data to exclude from the probability distribution predicted by the classifier those activities which are not context-consistent. In our experimental setup, this mechanism is based on the same ontology used by the Context-Aware Data Association module.

Indeed, as it is possible to observe in Fig. 2, our ontology also contains axioms about the relationships between context data and activities.

MICAR evaluates whether an activity *A* is context-consistent by adding to the ontology the factual observations about the current context $C(u)^t$ and $C_H^t$ and the fact that *u* is currently performing activity *A*. The non context-consistent activities are removed from the probability distribution *h*(*fv*), thus generating a refined probability distribution $h'(fv)$ over the possible *context-consistent* activities.

#### Example 3

Suppose that MICAR inferred that Alice is *watching television* with $60\%$ of probability, *eating* with the $30\%$ of probability and *setting up table* with the remaining $10\%$. According to our ontology, the *watching television* activity can be carried out only when: (a) the user is sitting (*user posture*), (b) the television is in the same user semantic position (*user and sensor position*) and (c) the television is turned on (*sensor status*). Suppose that Alice is sitting at the dining table while eating, while the television in the living room is turned on. Hence, *watching television* is not context-consistent for Alice considering how this activity is described in our knowledge base. The resulting re-normalized probability distribution of Alice in this case is $75\%$ *eating* and $25\%$ *setting up table*.

### Predictions aggregation

The goal of the Predictions Aggregation module is to detect activities that are jointly performed by multiple residents. For the sake of this work, we assume that a group activity occurs when two or more residents perform the same activity *A* in the same smart-home location *l* during the same time interval.

Note that this module covers the case where different residents start to perform the group activity at different times. For instance, consider a scenario where Alice watches the television and then eats, while Bob sets up the table and then eats. Bob starts eating 5 min before Alice. The Perdictions Aggregation module would detect the group activity *eating* only when both Alice and Bob are eating. Moreover, the assumption on the semantic locations allows MICAR to capture the scenario where the same type of ADL is performed by different residents in different rooms (e.g., Alice is watching TV in the living room, while Bob is watching TV in the bedroom).

In order to derive group ADLs, MICAR analyses the activities predicted for each resident by the single-inhabitant classifier and the residents’ location during their execution. In particular, for each resident, the output of the classifier is processed in real-time to keep track of *stable activities predictions*. Given a resident *u*, a *stable prediction* *S*(*u*, *A*, *L*, [*ti*, *tj*]) is generated from a sequence of consecutive feature vectors of *u* classified with the same activity *A* performed in the location *l* during the time interval [*ti*, *tj*]. In order to be considered stable, during [*ti*, *tj*] the confidence on *A* should be higher than a threshold *c* for at least *t* times. In our experiments, we empirically determined $c = 0.75$ and $t = 3$.

Two residents $u_i$ and $u_j$ jointly perform an activity *A* if there exists two stable predictions $S(u_1,A,L,[t_i,t_j])$ and $S(u_2,A,L,[t_l,t_k])$ such that $[t_i,t_j]$ and $[t_l,t_k]$ temporally overlap. The overlap between the time intervals determine the duration of the joint activity. Clearly, this process works in a similar way for more than two residents.

Note that the specific aggregation approach that should be adopted depends on the nature of the dataset and the specific target application. For instance, in a real-world scenario, more users could perform a collaborative activity while playing the same online multiplayer videogame in different locations of the smart-home, using different computers. For the sake of this work, we only target group activities that occur in the same location.

### Prediction confidence evaluation

While MICAR uses context-aware prediction refinement to mitigate classification errors, the system may still be uncertain on the refined prediction. The Prediction confidence evaluation module takes advantage of a semi-supervised strategy based on active learning to trigger a query to the resident when the confidence on the refined prediction is below a certain threshold. Since this evaluation is performed on the output of a single-inhabitant classifier, our active learning strategy is not targeted to joint activities.

For each $h^{\prime }(fv)$ generated by the prediction refinement module, we compute uncertainty based on the entropy of the probability distribution:

$$\begin{aligned} H(h^{\prime }(fv)) = \sum _i p^{\prime }_{A_i} \log \frac{1}{p^{\prime }_{A_i}} \end{aligned}$$

where $p^{\prime }_{A_i}$ is the refined probability distribution related to activity $A_i$. Note that the entropy measure is commonly used to compute the uncertainty in active learning [43]. When the entropy is higher than a threshold $\pi$, we assume that the system is uncertain about the activity currently performed by *u*. Hence, an active learning process is started, and MICAR asks to *u* a feedback about the activity that she was actually performing. For the sake of usability, only a few alternatives among the most likely activities are proposed. In our experiments we determined $\pi = 0.6$

The feedback is then considered to update the incremental activity recognition classifier as a new labeled data sample. MICAR updates the classifier when a batch of *w* feedback is obtained by the residents. In our experiments, we empirically determined $w=32$ to balance the trade-off between convergence rapidity and recognition stability. For the sake of this work, the feedback from each resident contributes to updating the same single-inhabitant classifier that is used for every resident.

Active learning generally leads to good recognition rates for activity recognition [10]. However, a high number of queries negatively impacts the user experience. Since we periodically update the model with a batch of feedback, MICAR can potentially maintain the same uncertainty for consecutive feature vectors until the model is not updated. In order to mitigate this problem, MICAR implements a novel active learning strategy based on caching. In particular, for each user *u*, MICAR stores the latest uncertainty prompted to *u* as the set of the two most likely activities $\{A_i$, $A_j\}$ in the probability distribution 3, and the feedback provided by *u*. Hence, if the same uncertainty occurs multiple times within a short time period for a specific user, MICAR does not trigger additional queries and it uses the last feedback provided by *u* to update the classifier. When a new uncertainty occurs, MICAR overwrites the user’s cache. After a certain amount of time, defined by the constant *CACHE_TTL*, MICAR invalids the cache. The MICAR’s active learning approach is described in detail in Algorithm 1.

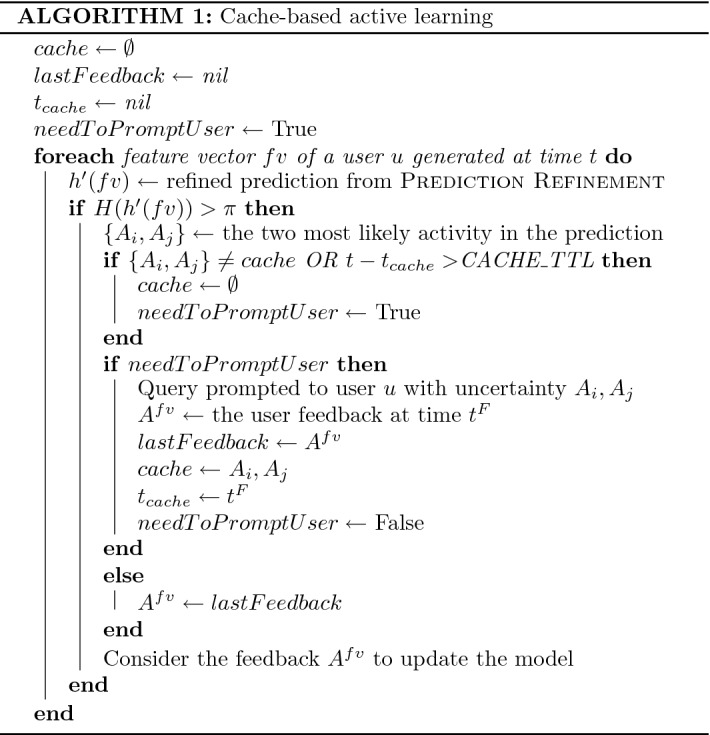



## Evaluation

### The dataset

**Fig. 3:**
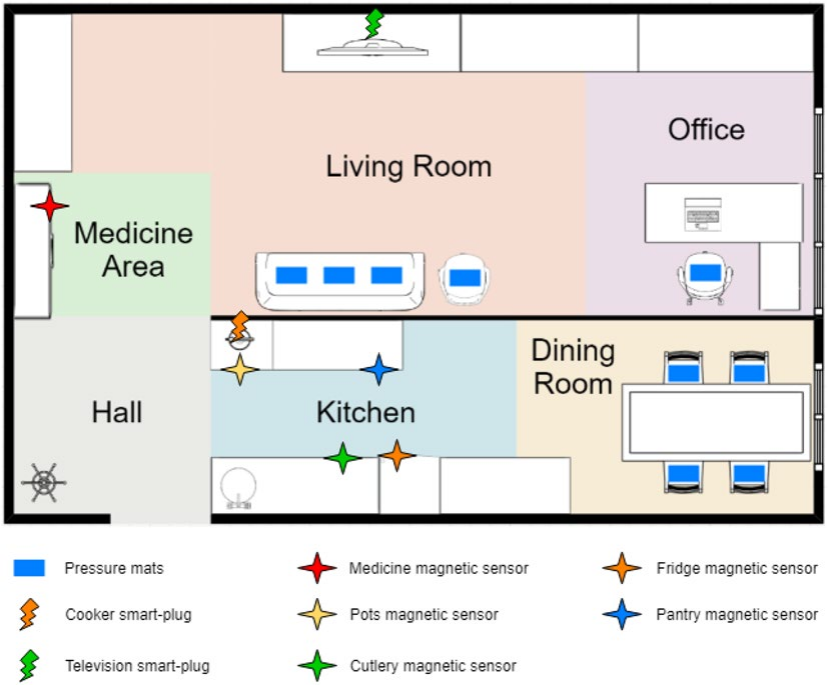
The simulated smart-home during the dataset collection process

In order to adequately evaluate MICAR, we collected a novel dataset (called MARBLE) in our smart-home lab. This dataset is publicly available [4]. To the best of our knowledge, there are no other publicly available multi-inhabitant ADLs datasets that combine wearable and environmental sensor data to provide the context information required by MICAR. As depicted in Fig. 3, we equipped the smart-home lab with several environmental sensors: magnetic sensors to detect the opening and closing events of drawers (e.g., fridge, medicine cabinet), pressure mat sensors to detect when residents are sitting on chairs/sofa, and plug sensors to detect the usage of home appliances (e.g., TV, electric cooker). To monitor phone call activities, the residents carried an Android smartphone in their pockets running a dedicated application to detect starting and ending events of incoming and outgoing phone calls. The residents were also wearing a smartwatch^4^ to collect data from inertial sensors (i.e., accelerometer, gyroscope, and magnetometer). We also deployed a positioning infrastructure composed of BLE beacons and WiFi APs. However, since indoor positioning is orthogonal to ADLs recognition, in our experiments we considered the ground truth about the position of each resident within the smart home. The smart-home lab was divided into 6 semantic locations: *Dining Room*, *Hall*, *Kitchen*, *Living Room*, *Medicine Area*, *Office*.

**Fig. 4:**
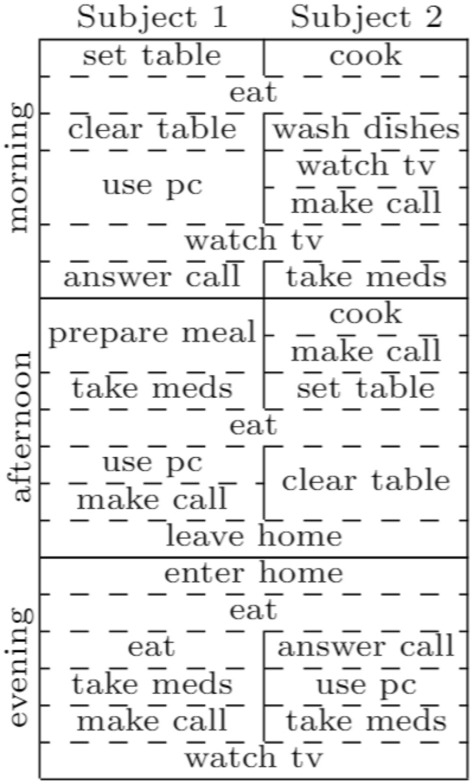
A scenario involving two inhabitants

The dataset includes 13 ADLs: *Answering Phone*, *Clearing Table*, *Cooking*, *Eating*, *Getting In*, *Getting Out*, *Making Phone Call*, *Preparing Cold Meal*, *Setting Up Table*, *Taking Medicines*, *Using PC*, *Washing Dishes*, and *Watching TV*. We recruited 12 volunteers not involved in this research. We instructed the volunteers about the sequence of activities they had to perform, but they were free to execute them in their way to increase the dataset variability. Our research team performed the annotations in real-time, thanks to cameras. We collected data considering both single- and multi-inhabitant scenarios, where a scenario describes a specific sequence of ADLs that the residents should perform. We designed several single-, 2- and 4-residents scenarios. For instance, Fig. 4 shows one of the 2-residents scenarios that we designed.

Each scenario was repeated several times by different volunteers. Overall, we acquired 12 instances of 4 single-inhabitant scenarios, 10 instances of 3 scenarios involving 2 subjects, and 10 other instances of 4 scenarios with 4 residents involved. Table 1 shows, for each ADL type, the amount of recorded labeled data in minutes, and the average duration in seconds, while Table 2 shows the recorded time and the average duration of single-, 2-, and 4-inhabitants scenarios. Note that, since we had time restrictions for data collection (due to the availability of volunteers), the execution time of each ADL was limited to a duration that in some cases does not reflect the actual time a person would need, but long enough to collect a significant amount of data. For instance, considering activities like *Eating* or *Cooking*, we asked our volunteers to perform them only for a few minutes.

**Table 1:** Statistics on labeled activities

	Recorded minutes	Average duration (s)
ANSWERING PHONE	68.5	70.8
CLEARING TABLE	38.3	42.6
COOKING	80.4	86.1
EATING	149.5	29.2
GETTING IN	18.8	12.8
GETTING OUT	13.6	16.4
MAKING PHONE CALL	63.4	56.8
PREPARING COLD MEAL	52.8	62.1
SETTING UP TABLE	53.5	42.8
TAKING MEDICINES	36.2	28.9
TRANSITION	273.4	13.4
USING PC	94.0	91.0
WASHING DISHES	54.4	51.8
WATCHING TV	266.7	98.2

**Table 2:** Statistics on the dataset scenarios

	Recorded minutes	Average duration (min)
Single-inhabitant scenarios	307.5	25.6
2-Inhabitants scenarios	315.5	31.5
4-Inhabitants scenarios	84.0	8.4

#### Evaluation methodology

In the following, we describe how we evaluate the recognition rate of MICAR. Since our semi-supervised activity recognition classifier is incremental, we adopt a well-known evaluation technique for stream learning algorithms [19]. We pre-train the classifier using labeled data from 2 subjects that only contributed to single-inhabitant scenarios. We use the remaining data to evaluate the evolution of the recognition rate and the number of questions triggered by active learning. We iterate over each data sample (i.e., feature vector), providing the classifier one instance of a scenario at a time. Within a scenario instance, the order of data samples provided to the classifier reflects the temporal order of data collection.

Each data sample is first classified using the current model. The ground truth and the classification output are stored for evaluation. Then, we apply the active learning strategy presented in Sect. 5.7 to determine if the query is needed. If this is the case, we use the data sample labeled with the ground truth to update the recognition model and we update the number of triggered questions.

In order to show the evolution of the classifier, we use a sliding window approach to periodically compute both the overall F1-score and the percentage of triggered questions. Each window contains 800 data samples, and we consider an overlap factor of $75\%$.

In order to achieve statistically robust results, the whole experiment is repeated 100 times, averaging the results. Moreover, at each repetition, we also randomly shuffle the order of the scenario instances that we provide to the classifier.

### Results

#### Recognition rate

**Fig. 5:**
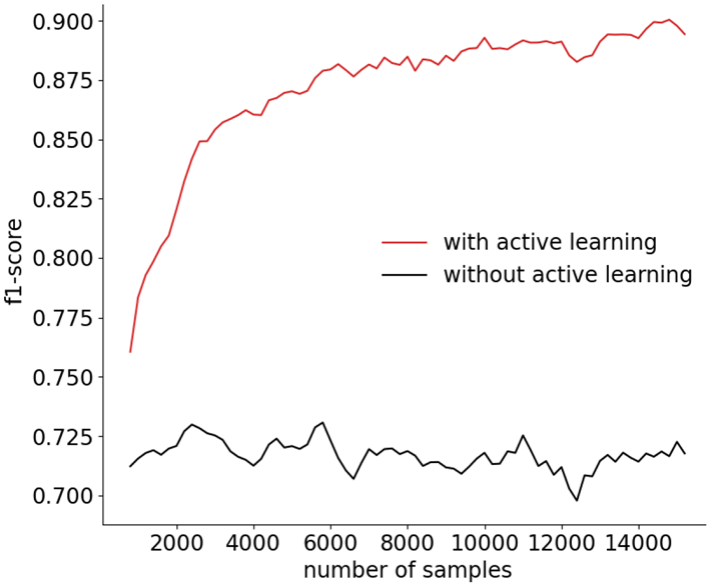
Evolution of the recognition rate of MICAR

In the following, we show results about the recognition rate of the Sensor-Based Activity Recognition module of MICAR, including the prediction refinement step. Figure 5 depicts the evolution of the recognition rate using the evaluation methodology presented above. Thanks to active learning, the recognition rate quickly converges to high values. Without active learning, the classifier (only pre-trained using data from 2 users) is never updated, and the f1-score is stable on low values.

**Fig. 6:**
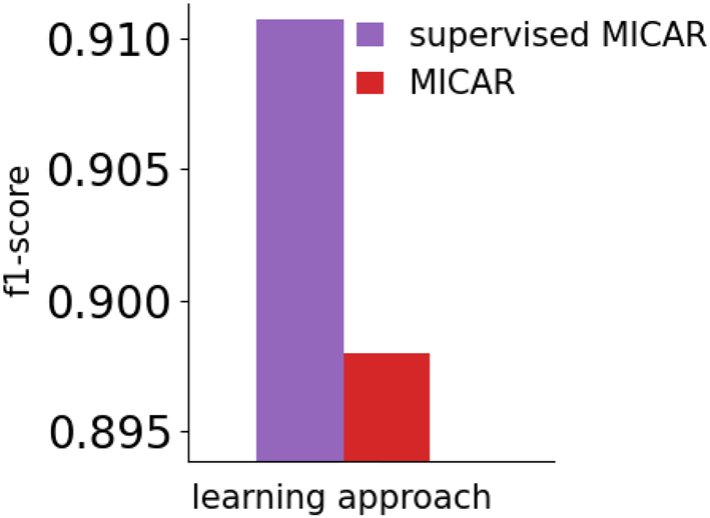
MICAR vs a fully supervised approach

Figure 6 compares the recognition rate reached by MICAR with the one obtained by a supervised version of MICAR (i.e., with full availability of labeled data and without active learning). We will refer to this approach as *Supervised* MICAR.

We computed the F1-score of MICAR by considering the mean of the F1-scores obtained on the last four windows (see Fig. 5). On the other hand, we computed the F1-score of *Supervised* MICARusing a leave-one-scenario-out cross-validation approach. At each fold, we considered a specific instance of a scenario as the test set, while the data of all the remaining scenario instances as the training set. To make our validation robust, we also removed from the training set: (1) data related to the other instances of the same scenario in the test set, (2) data of the subjects in the test set. We observed that the recognition rate of MICAR is only $\approx 1\%$ behind the one reached by *Supervised* MICAR, with the great advantage of requiring a limited amount of labeled data. In Sect. 6.2.2 we show results about the number of active learning queries triggered by MICAR.

**Fig. 7:**
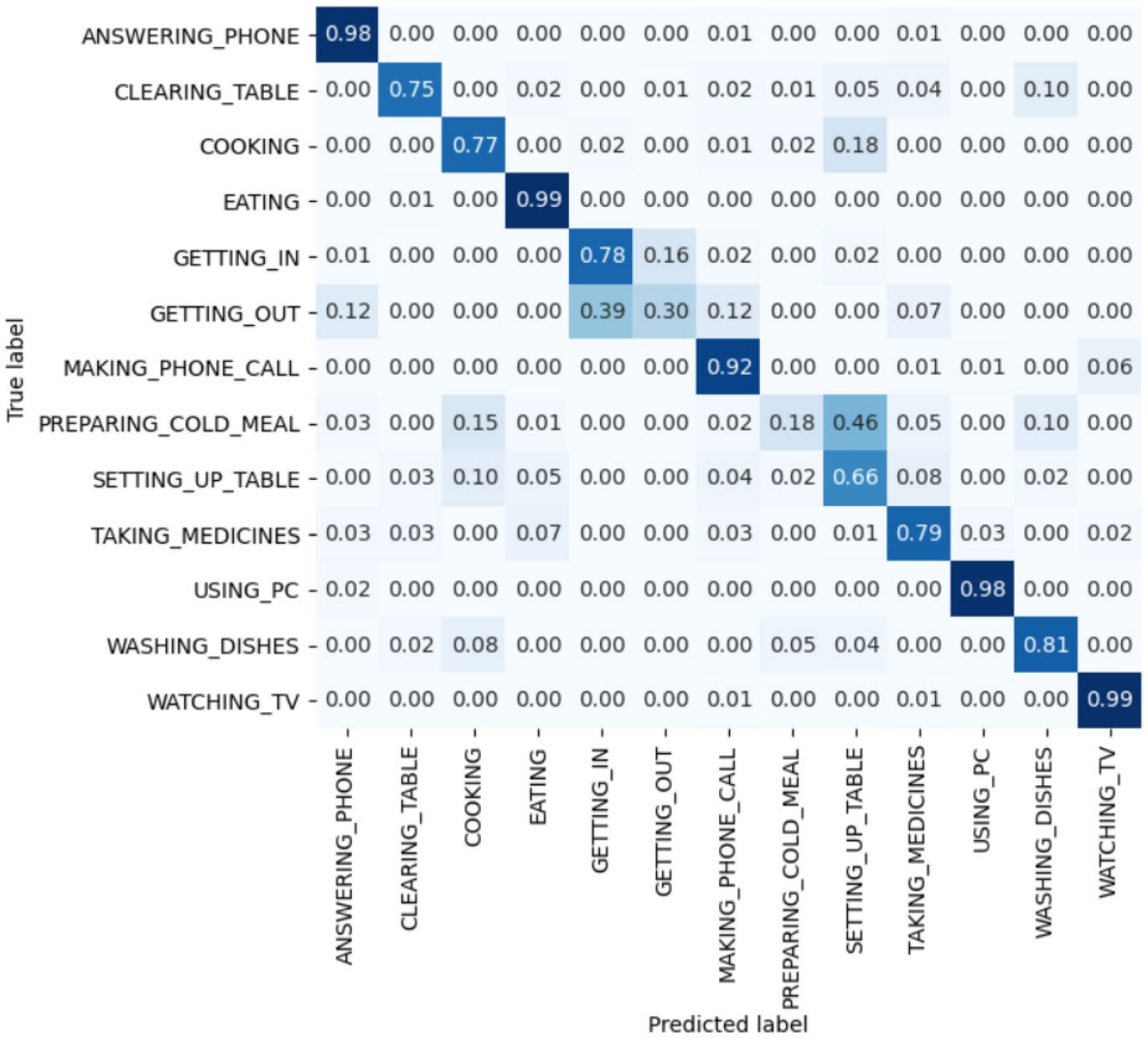
Confusion matrix

Figure 7 shows the confusion matrix generated by MICAR.

Activities like *watching TV* and *using pc* are recognized with a recall around $98\%$. Indeed, in our dataset, these activities are associated with specific semantic areas and environmental sensors that uniquely characterize them. For example, *watching TV* can only be performed in the *living room* triggering the smart-plug sensor connected to the television.

On the other hand, those activities that are not uniquely characterized by available context data exhibit a lower recognition rate. For example, the activities that can be performed by standing in the kitchen (e.g., *preparing a cold meal*, *setting up the table*, and *cooking*) are often confused between them since they trigger similar sensors. Nonetheless, *cooking* still reaches good recognition rates thanks to the plug sensor that detects the electrical stove usage. Also, we observed that the activities *clearing table* and *washing dishes* are well-recognized even if they are associated with context information similar to the above-mentioned kitchen-based activities. Considering *clearing table*, this is likely due to the capability of recurrent layers to capture the temporal relationships between activities, since *clearing table* is often performed after *eating*. Considering *washing dishes*, inertial sensor data capture the gestures that uniquely characterize the activity. MICAR also confuses *getting in* and *getting out* activities due to their similar patterns. The remaining activities are well-recognized by MICAR.

#### Effectiveness of active learning

Besides the recognition rate, a fundamental aspect is the number of questions triggered by active learning due to its direct impact on user experience. Figure 9 shows that the percentage of active learning questions quickly converges to low values (below $5\%$) with a decreasing trend (i.e., the system asks fewer and fewer questions over time).

**Fig. 8:**
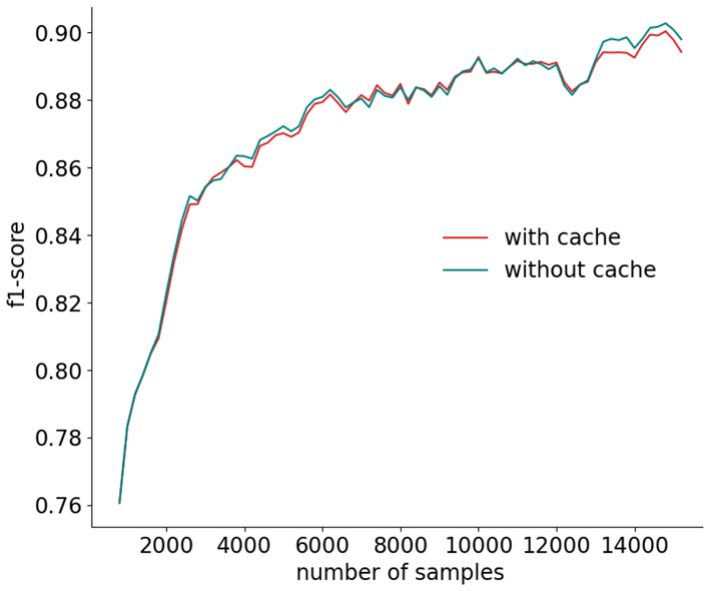
Impact of the cache on the evolution of the recognition rate

**Fig. 9:**
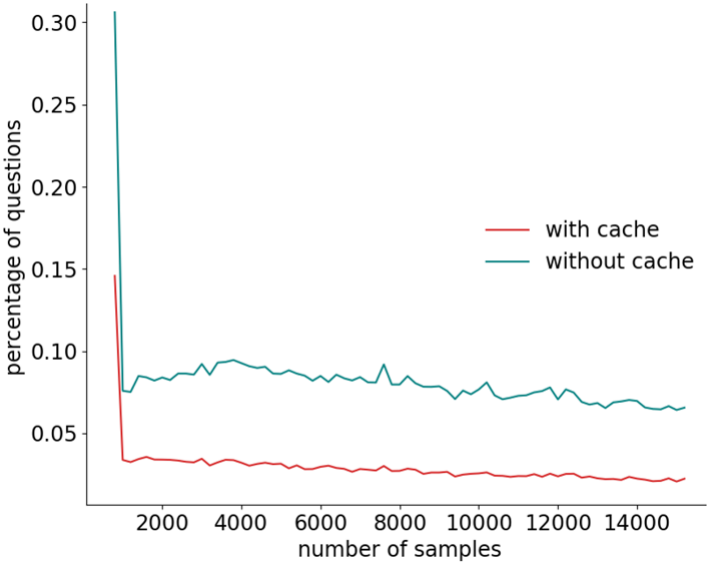
Impact of the cache on the evolution of the percentage of active learning queries

Figures 8 and 9 also compare our cache-based approach described in Sect. 5.7 with respect to a traditional method that does not use a cache (i.e., a query is triggered every time there is an uncertainty).

We observed that the recognition rate of our cache-based method is almost identical to the one reached by a traditional approach, while the percentage of questions is dramatically lower. We also observed that the cache was used by MICAR $66\%$ of the times there was an uncertainty. This is due to the fact that MICAR updates the classifier with a batch-based approach. Hence, since the model update is delayed, the classifier often has the same uncertainty on consecutive feature vectors.

#### Context-aware data association

**Fig. 10:**
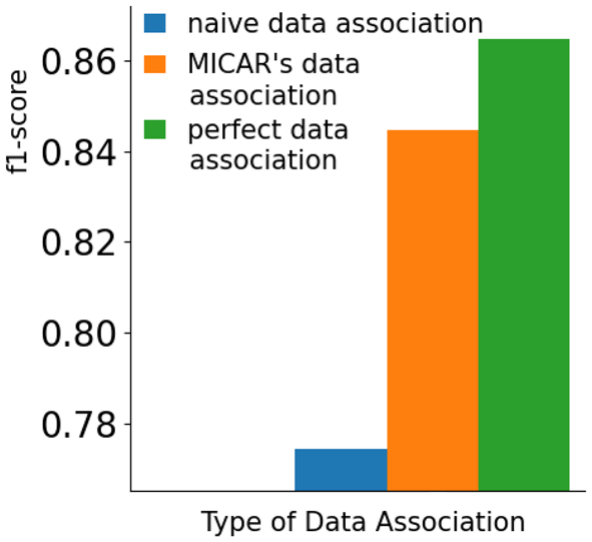
The recognition rate obtained by our data association approach with respect to a naive solution and an ideal solution

**Fig. 11:**
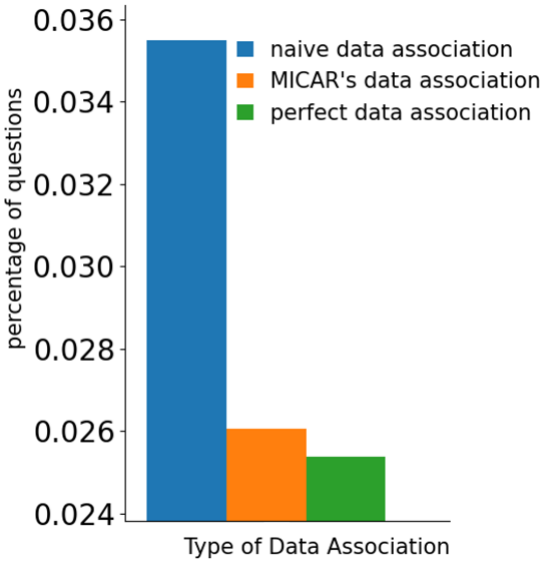
The percentage of questions obtained by our data association approach with respect to a naive solution and an ideal solution

Figures 10 and 11 show the effectiveness of our data association method compared with two alternatives. The first is called *naive data association*, and it simply assigns each environmental sensor event to every resident in the home, independently from context data. The second one is called *perfect data association*, and it assigns each environmental sensor event to the correct user by using the ground truth. Clearly, *perfect data association* is an ideal approach that cannot be implemented in practice, and we consider it as an upper bound. Note that, to better highlight the impact of data association, we show the results that we obtained without prediction refinement.

The data association strategy of MICAR significantly outperforms in terms of f1-score the *naive data association* approach ($+6\%$). At the same time, our solution is only $2\%$ behind a *perfect data association*. These results suggest that our data association approach is accurate. Considering the number of active learning queries, the data association strategy of MICAR triggers a reduced number of queries than the *naive data association* solution, reaching very close results to *perfect data association*.

#### Prediction refinement

**Fig. 12:**
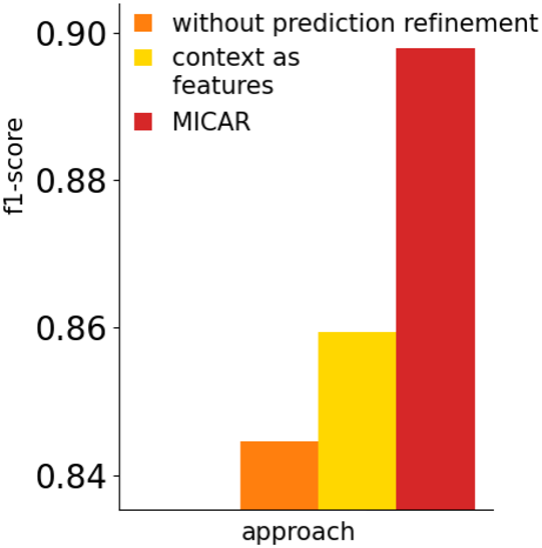
The impact of our prediction refinement approach on the recognition rate

**Fig. 13:**
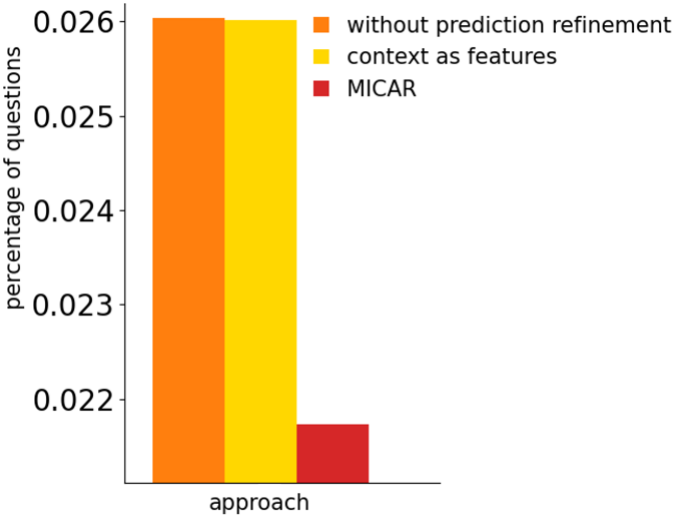
The impact of our prediction refinement approach on the percentage of questions

Figures 12 and 13 show the impact of the prediction refinement module in refining the classification mistakes. We compare our method with two alternatives: *without prediction refinement* and *context as features*. The first is MICAR without the prediction refinement module. Hence, the classification output is not refined using context. On the other hand, the *context as features* approach considers context information as additional features in the machine learning process, instead of processing them with a knowledge-based approach after classification.

Our results show that context data significantly improves the recognition rate. Indeed, the *without context* approach reaches the lowest F1-score. Moreover, MICAR outperforms the *context as features* solution ($+4\%$). This is due to the fact that ADLs can be performed in many different context situations. Considering context as features makes the learning task more complex, thus requiring more labeled data. Moreover, our knowledge-based approach is more flexible since new context information can be added dynamically to the ontology, while the machine learning classifier should be re-trained from scratch if new features need to be considered.

Considering active learning queries, MICAR outperforms both approaches. Indeed, by discarding the context-inconsistent activities, MICAR often increases its confidence on the remaining activities, thus reducing the percentage of triggered questions.

#### Predictions aggregation

Finally, we quantitatively evaluate the effectiveness of the predictions aggregation module in detecting jointly performed activities. For the sake of this evaluation, we only considered data from $2-$ and $4-$residents scenarios.

**Fig. 14:**
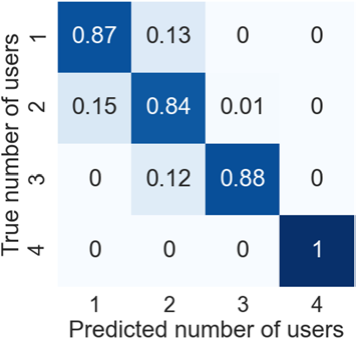
Confusion matrix on the number of users attributed to group activities

We used the method proposed in Sect. 5.6 to compute group activities both on the classification output as well as on the ground truth. Figure 14 shows a confusion matrix that reveals MICAR’s accuracy in detecting the correct number of users that are jointly performing an activity. Let $\langle A, U, [t_i,t_j] \rangle$ be a detected group activity where *A* is the joint activity, *U* is the set of residents jointly performing *A*, and $[t_i,t_j]$ is the time interval of the group activity. Similarly, let $\langle A^\star , U^\star , [t_l,t_k] \rangle$ be a ground truth group activity where $A^\star$ is the joint activity, $U^\star$ is the set of residents performing $A^\star$, and $[t_l,t_k]$ is the time interval of the ground truth group activity. We compare a predicted group activity and a ground truth group activity when $A = A^*$ and $[t_i,t_j]\cap [t_l,t_k]\ne \emptyset$ (i.e., the activity is the same and they temporally overlap). Hence, in this evaluation we do not consider misclassifications, that are already captured by the results reported in Fig. 7.

We consider a true positive when $U = U^\star$ (i.e., the set of users is exactly the same). We consider a false positive when $U \supset U^\star$ (i.e., the predicted group activity involves a higher number of residents w.r.t. the ground truth). Finally, a false negative occurs when $(U \cap U^\star ) \subset U^\star$ (i.e., only a subset of the residents in the ground truth is actually in the prediction).

From the confusion matrix, we observed that individual activities are sometimes detected as 2-residents activities. This is probably due to mistakes in data association. For instance, if Alice is preparing a salad in the kitchen while Bob is cooking pasta in the same room, the electric cooker event could be mistakenly assigned to both the residents. Hence, MICAR could detect that Alice and Bob are cooking together over a certain interval of time.

Group activities are sometimes detected with a lower number of users with respect to the ground truth. This could happen when MICAR performs a miss-classification for a subset of users in the group. For example, suppose that Alice washed the dishes from *t*0 to *t*3, while Bob was clearing the table in the same time interval. From *t*4 to *t*6 they watched television together. MICAR may correctly predict Bob’s activity while it may mistakenly detect that Alice washed the dishes from *t*0 to *t*4 and that she started to watch the television with Bob at *t*5. In this case, we count a true positive (Alice and Bob watched the television together from *t*5 to *t*6), but also a false negative (Bob individually watched the television from *t*4 to *t*5).

The accurate recognition rate for 4-resident activities is due to the fact that, in our dataset, only ADLs that are easy to detect (like eating and watching tv) are performed in this setting.

## Discussion

### Acceptability and privacy issues

MICAR is an ADL recognition system that continuously records the behavior of the residents in their daily life. Considering the application of our framework for healthcare applications, it may be perceived as a component of a therapy, hence it is more likely accepted with respect to other solutions. Moreover, MICAR does not consider intrusive devices like microphones and cameras. However, the data collected by MICAR are sensitive, and privacy measures should be considered in order to manage them. In our vision, the MICARalgorithms should run on the smart-home gateway and detailed sensor data should not be accessible from outside. In order to release information to healthcare stakeholders (e.g., clinicians), there are several solutions. Among them, aggregated ADLs data can be outsourced in an encrypted form to a cloud server. By relying on searchable encryption, it is possible to outsource encrypted data and, at the same time, to allow clinicians to perform queries on encrypted data [52]. We are currently investigating the effectiveness of such a solution.

Active learning may also be considered invasive and ethically inappropriate. Indeed, each query is an interruption to the daily life of a resident. Hence, active learning queries may not be acceptable if too frequent or if they are prompted at inappropriate times. While we show that the number of queries generated by MICAR is low and their frequency decreases quickly, in future work we will investigate a context-aware strategy in charge of prompting active learning queries based on the resident’s context, interrupting her only when appropriate.

### Personalization

Personalization is an important aspect for accurate ADLs recognition [18]. This is also true for data association. Indeed, we believe that the additional personal context of the residents may further improve data association. For instance, each resident may have specific habits and routines, also depending on the role in the home (e.g., caregiver, elderly woman, elderly man). For instance, if the caregiver and an elderly subject are at the same time in the kitchen while the stove is being turned on, the caregiver is more likely the one triggering this event. This high-level information can be considered as the system’s prior knowledge or, alternatively, it can be automatically derived using pattern mining approaches that learn typical routines of each subject.

We also believe that the personal agenda of each resident may help in providing hints about data association (e.g., if Alice has a dentist appointment in 20 min, she is more likely the one that is opening the door to leave home). In future work, we will investigate how to improve personalization aspects in MICAR.

### Need for real-world experiments

A limitation of this work is that experiments are conducted using a public dataset acquired in a controlled setting. Experiments using real-world datasets are needed to better assess the effectiveness of our approach. We plan to perform this evaluation in the future, in the context of research projects related to healthcare.

Moreover, for the sake of this work, we did not consider the remote control of smart devices. Considering the specific sensors that we adopted in our experimental setup, only the smart plugs (controlling the TV and the stove) could actually be remotely controlled. Indeed, other devices like magnetic and mat sensors require physical interaction with the subject.

The remote control of smart devices introduces new challenges as we illustrate in the following example.

#### Example 4

Alice is in the kitchen, while Bob is in the living room. Since Bob intends to prepare some food in the next few minutes, he decides to remotely turn on the oven to warm it up while he is still in the living room. MICAR mistakenly associates the event *Turning ON oven* to Alice, since she is the one actually in the kitchen.

We believe that the data association strategy of MICAR can be extended to consider the remote activation of smart devices. For instance, when a resident controls a device by using its personal smartphone, the association is straightforward (i.e., the smartphone directly identifies the resident). However, smart devices may be also controlled using voice-based home assistants. In this scenario, a possible solution is to identify the users through the voice captured by the microphone.

Note that, considering Example 4, when Bob is turning on the oven from the living room, it is not clear if this event should be actually associated with him. Indeed, it is not trivial to determine what ADL classes the system should recognize when events related to remote control of smart devices are detected. We will investigate this direction in future work.

## Conclusion and future work

In this paper, we presented MICAR: a novel Multi-Inhabitant semi-supervised and Context-aware Activity Recognition approach. Our method is based on a knowledge-based approach to perform data association. Moreover, MICAR relies on active learning to significantly reduce the amount of labeled data that is needed to train the activity classifier. Our results indicate that MICAR reaches similar results to a fully supervised multi-inhabitant approach, with a low number of triggered active learning queries. Moreover, the recognition rate reached by our data association approach is close to the one of an ideal perfect data association method based on ground truth.

MICAR has several limitations that we will address in future work. First, our knowledge-based approach requires a significant human engineering effort to generate an ontology that comprehensively captures all the relationships between contexts, sensor events, and activities. In future work, we will investigate how to populate the knowledge base in a semi-automatic way, by fetching context information from the web (e.g., textual information, images, etcetera). Another issue of ontological reasoning is its rigidity that cannot capture the intrinsic uncertainty of sensor data. Hence, will also investigate probabilistic knowledge-based approaches.

Another limitation is the assumption that a joint activity occurs only if the residents that are performing it are in the same semantic location. For instance, Alice and Bob may be jointly setting up the table before lunch. This ADL may be performed both in the kitchen (e.g., to retrieve silverware) and in the dining room (e.g., to prepare the table). In future work, we will investigate how to remove this assumption.

We also plan to evaluate in detail the acceptability of a system based on active learning. In [9], we proposed a hypothetical active learning interface for multi-inhabitant settings that also considers residents’ interruptibility. In future work, we will investigate in real-world deployments if this interface coupled with MICAR can have a positive impact on the perceived user experience.

Finally, we also plan to include more sophisticated context data (e.g., low-level activities, micro-localization at a finer granularity, social interaction between residents, temporal relationships between sensor events, etcetera) to further improve the accuracy of data association and prediction-refinement modules of MICAR.

## Footnotes

1 Note that here we assume that inhabitants that perform the same ADL in the same semantic place at the same time are actually jointly performing the ADL. This is indeed the case in our considered setting.

2 We performed several experiments considering up to 10 BLE beacons, but we observed interference problems when considering more than 5 beacons.

3 Note that we consider a set since the order of the two most likely activity is not relevant.

4 We used Huawei Sport 2 and other brands with similar features.
